# 4-(Methyl­sulfan­yl)-2-(*p*-toluene­sulfonamido)butanoic acid

**DOI:** 10.1107/S1600536808005035

**Published:** 2008-03-12

**Authors:** Li Wang, Zheng Liu, Yong Liao Wang

**Affiliations:** a Key Laboratory of Non-ferrous Metal Materials and Processing Technology, Department of Materials and Chemical Engineering, Ministry of Education, Guilin University of Technology, Guilin 541004, People’s Republic of China

## Abstract

In the title compound, C_12_H_17_NO_4_S_2_, the carboxyl groups link the mol­ecules into centrosymmetric dimers through O—H⋯O hydrogen bonds. An N—H⋯O hydrogen bond between the NH group of the l-methio­nine unit and a neighbouring carboxyl group, together with a complementary C—H⋯O contact to one O atom of the sulfonyl group, link the dimers into one-dimensional chains along the crystallographic *b* axis.

## Related literature

The title compound is closely related to the previously reported *N*-tosyl-l-glutamic acid (Zachara *et al.*, 2005 ▶).
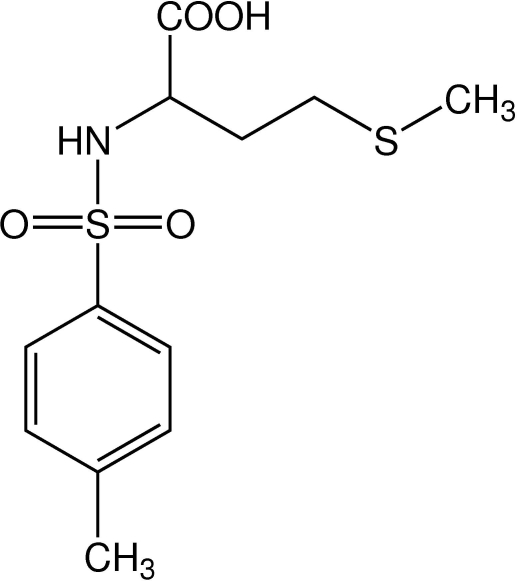


## Experimental

### 

#### Crystal data

C_12_H_17_NO_4_S_2_
*M* *_r_* = 303.39
Monoclinic, 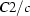

*a* = 33.121 (7) Å
*b* = 5.6531 (11) Å
*c* = 17.278 (4) Å
β = 116.62 (3)°
*V* = 2892.2 (10) Å^3^
*Z* = 8
Mo *K*α radiation
μ = 0.38 mm^−1^
*T* = 293 (2) K
0.43 × 0.28 × 0.22 mm

#### Data collection

Bruker APEXII CCD diffractometer
Absorption correction: multi-scan (*SADABS*; Bruker, 2001 ▶) *T*_min_ = 0.855, *T*_max_ = 0.922
6139 measured reflections
2689 independent reflections
2055 reflections with *I* > 2σ(*I*)
*R*_int_ = 0.028

#### Refinement

*R*[*F* ^2^ > 2σ(*F* ^2^)] = 0.043
*wR*(*F* ^2^) = 0.119
*S* = 1.00
2689 reflections
179 parameters
H atoms treated by a mixture of independent and constrained refinement
Δρ_max_ = 0.30 e Å^−3^
Δρ_min_ = −0.25 e Å^−3^

### 

Data collection: *APEX2* (Bruker, 2004 ▶); cell refinement: *SAINT-Plus* (Bruker, 2001 ▶); data reduction: *SAINT-Plus*; program(s) used to solve structure: *SHELXS97* (Sheldrick, 2008 ▶); program(s) used to refine structure: *SHELXL97* (Sheldrick, 2008 ▶); molecular graphics: *SHELXTL* (Sheldrick, 2008 ▶); software used to prepare material for publication: *SHELXTL*.

## Supplementary Material

Crystal structure: contains datablocks global, I. DOI: 10.1107/S1600536808005035/bi2276sup1.cif

Structure factors: contains datablocks I. DOI: 10.1107/S1600536808005035/bi2276Isup2.hkl

Additional supplementary materials:  crystallographic information; 3D view; checkCIF report

## Figures and Tables

**Table 1:** Hydrogen-bond geometry (Å, °)

*D*—H⋯*A*	*D*—H	H⋯*A*	*D*⋯*A*	*D*—H⋯*A*
O4—H4*A*⋯O3^i^	0.82	1.85	2.672 (3)	174
N1—H1⋯O4^ii^	0.81 (3)	2.62 (3)	3.405 (3)	163 (2)
C4—H4⋯O1^iii^	0.98	2.25	3.165 (3)	155

Symmetry codes: (i) 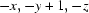; (ii) 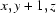; (iii) 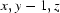.

**Fig. 1.**
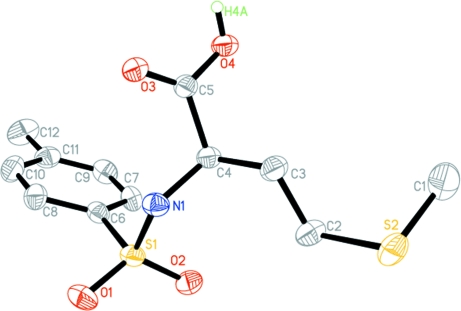
The molecular structure of the title compound showing 30% probability displacement ellipsoids for non-H atoms. H atoms bound to C and N are omitted.

**Fig. 2.**
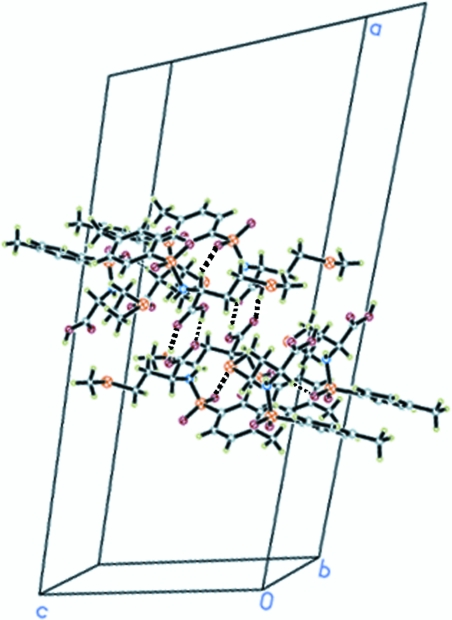
Partial packing diagram showing a hydrogen-bonded chain running along the b axis.

## supplementary crystallographic information

### Comment

The title compound (Fig. 1) was synthesized from 4-toluenesulfonyl chloride and *L*-methionine. It is closely related to the previously reported *N*-tosyl-*L*-glutamic acid (Zachara *et al.*, 2005).

### Experimental

A solution of *L*-methionine (0.005 mmol) and NaOH (0.015 mmol) in water (15 ml) was added to an ethanol solution of 4-toluenesulfonyl chloride (0.005 mmol). After stirring at 348 K for 40 min, crystals of the title compound were obtained by slow evaporation of the reaction mixture at room temperature.

### Refinement

H atoms bound to C or O atoms were placed geometrically with C—H = 0.93–0.97 Å or O—H = 0.82 Å and refined as riding with *U*_iso_(H) = 1.2 or 1.5*U*_eq_(C/O). The H atom of the NH group was located in a difference Fourier map and refined with an isotropic displacement parameter, without restraint.

### Crystal data

**Table:**

C_12_H_17_NO_4_S_2_	*F*_000_ = 1280
*M**_r_* = 303.39	*D*_x_ = 1.394 Mg m^−^^3^
Monoclinic, *C*2/*c*	Mo *K*α radiation λ = 0.71073 Å
Hall symbol: -C 2yc	Cell parameters from 2689 reflections
*a* = 33.121 (7) Å	θ = 2.8–25.5º
*b* = 5.6531 (11) Å	µ = 0.38 mm^−^^1^
*c* = 17.278 (4) Å	*T* = 293 (2) K
β = 116.62 (3)º	Block, colorless
*V* = 2892.2 (10) Å^3^	0.43 × 0.28 × 0.22 mm
*Z* = 8	

### Data collection

**Table:**

Bruker APEXII CCD diffractometer	2689 independent reflections
Radiation source: fine-focus sealed tube	2055 reflections with *I* > 2σ(*I*)
Monochromator: graphite	*R*_int_ = 0.028
*T* = 293(2) K	θ_max_ = 25.5º
φ and ω scans	θ_min_ = 2.8º
Absorption correction: multi-scan(SADABS; Bruker, 2001)	*h* = −40→30
*T*_min_ = 0.855, *T*_max_ = 0.922	*k* = −5→6
6139 measured reflections	*l* = −17→20

### Refinement

**Table:**

Refinement on *F*^2^	Secondary atom site location: difference Fourier map
Least-squares matrix: full	Hydrogen site location: inferred from neighbouring sites
*R*[*F*^2^ > 2σ(*F*^2^)] = 0.043	H atoms treated by a mixture of independent and constrained refinement
*wR*(*F*^2^) = 0.119	*w* = 1/[σ^2^(*F*_o_^2^) + (0.0619*P*)^2^ + 2.0643*P*] where *P* = (*F*_o_^2^ + 2*F*_c_^2^)/3
*S* = 1.00	(Δ/σ)_max_ < 0.001
2689 reflections	Δρ_max_ = 0.30 e Å^−^^3^
179 parameters	Δρ_min_ = −0.25 e Å^−^^3^
Primary atom site location: structure-invariant direct methods	Extinction correction: none

### Special details

**Table:**

Geometry. All e.s.d.'s (except the e.s.d. in the dihedral angle between two l.s. planes) are estimated using the full covariance matrix. The cell e.s.d.'s are taken into account individually in the estimation of e.s.d.'s in distances, angles and torsion angles; correlations between e.s.d.'s in cell parameters are only used when they are defined by crystal symmetry. An approximate (isotropic) treatment of cell e.s.d.'s is used for estimating e.s.d.'s involving l.s. planes.
Refinement. Refinement of *F*^2^ against ALL reflections. The weighted *R*-factor *wR* and goodness of fit *S* are based on *F*^2^, conventional *R*-factors *R* are based on *F*, with *F* set to zero for negative *F*^2^. The threshold expression of *F*^2^ > σ(*F*^2^) is used only for calculating *R*-factors(gt) *etc*. and is not relevant to the choice of reflections for refinement. *R*-factors based on *F*^2^ are statistically about twice as large as those based on *F*, and *R*- factors based on ALL data will be even larger.

### Fractional atomic coordinates and isotropic or equivalent isotropic displacement parameters (Å^2^)

**Table:**

	*x*	*y*	*z*	*U*_iso_*/*U*_eq_	
C1	0.05669 (12)	0.1441 (6)	−0.2992 (2)	0.0798 (10)	
H1A	0.0814	0.0695	−0.2516	0.120*	
H1B	0.0514	0.0655	−0.3521	0.120*	
H1C	0.0300	0.1343	−0.2906	0.120*	
C2	0.09539 (9)	0.5313 (5)	−0.19259 (17)	0.0532 (7)	
H2A	0.1186	0.4170	−0.1596	0.064*	
H2B	0.1098	0.6843	−0.1860	0.064*	
C3	0.06194 (8)	0.5443 (5)	−0.15563 (16)	0.0500 (7)	
H3A	0.0477	0.3909	−0.1619	0.060*	
H3B	0.0385	0.6573	−0.1890	0.060*	
C4	0.08314 (7)	0.6170 (4)	−0.05971 (14)	0.0386 (5)	
H4	0.1099	0.5190	−0.0273	0.046*	
C5	0.04899 (8)	0.5711 (4)	−0.02597 (14)	0.0392 (5)	
C6	0.16839 (7)	0.8595 (4)	0.11546 (15)	0.0405 (5)	
C7	0.19182 (8)	0.6522 (5)	0.14801 (16)	0.0480 (6)	
H7	0.1967	0.5472	0.1117	0.058*	
C8	0.16108 (9)	1.0154 (5)	0.17034 (18)	0.0512 (7)	
H8	0.1452	1.1554	0.1488	0.061*	
C9	0.20788 (9)	0.6019 (5)	0.23483 (18)	0.0538 (7)	
H9	0.2235	0.4612	0.2563	0.065*	
C10	0.17754 (9)	0.9604 (5)	0.25668 (18)	0.0586 (7)	
H10	0.1724	1.0644	0.2930	0.070*	
C11	0.20148 (8)	0.7549 (5)	0.29100 (17)	0.0526 (7)	
C12	0.22040 (10)	0.7014 (7)	0.38595 (19)	0.0769 (10)	
H12A	0.2242	0.5336	0.3948	0.115*	
H12B	0.2000	0.7580	0.4074	0.115*	
H12C	0.2491	0.7783	0.4164	0.115*	
N1	0.09655 (7)	0.8649 (4)	−0.04861 (13)	0.0429 (5)	
H1	0.0819 (9)	0.958 (5)	−0.0360 (18)	0.051 (8)*	
O1	0.15126 (6)	1.1856 (3)	0.00061 (12)	0.0545 (5)	
O2	0.17393 (5)	0.7911 (3)	−0.02689 (11)	0.0520 (5)	
O3	0.02802 (6)	0.7289 (3)	−0.01265 (11)	0.0479 (4)	
O4	0.04342 (6)	0.3447 (3)	−0.01734 (12)	0.0499 (5)	
H4A	0.0204	0.3249	−0.0115	0.075*	
S1	0.149864 (19)	0.93464 (11)	0.00597 (4)	0.0414 (2)	
S2	0.07010 (3)	0.44879 (15)	−0.30518 (4)	0.0630 (3)	

### Atomic displacement parameters (Å^2^)

**Table:**

	*U*^11^	*U*^22^	*U*^33^	*U*^12^	*U*^13^	*U*^23^
C1	0.100 (3)	0.073 (2)	0.069 (2)	0.007 (2)	0.0400 (19)	−0.0020 (17)
C2	0.0484 (14)	0.072 (2)	0.0443 (14)	0.0011 (13)	0.0249 (12)	0.0054 (13)
C3	0.0437 (14)	0.0659 (18)	0.0441 (14)	−0.0064 (12)	0.0231 (11)	−0.0046 (12)
C4	0.0380 (12)	0.0417 (14)	0.0382 (12)	−0.0031 (10)	0.0190 (10)	0.0019 (10)
C5	0.0424 (13)	0.0435 (15)	0.0326 (12)	−0.0045 (11)	0.0175 (10)	−0.0006 (10)
C6	0.0353 (12)	0.0368 (13)	0.0474 (13)	−0.0032 (10)	0.0167 (10)	−0.0014 (11)
C7	0.0503 (14)	0.0377 (14)	0.0509 (15)	0.0017 (12)	0.0182 (12)	−0.0033 (11)
C8	0.0529 (15)	0.0439 (16)	0.0607 (17)	0.0093 (12)	0.0290 (13)	0.0011 (12)
C9	0.0474 (15)	0.0459 (16)	0.0599 (17)	0.0045 (12)	0.0168 (13)	0.0079 (13)
C10	0.0564 (16)	0.068 (2)	0.0570 (17)	0.0012 (14)	0.0301 (14)	−0.0105 (14)
C11	0.0374 (13)	0.0666 (19)	0.0518 (15)	−0.0045 (13)	0.0182 (11)	0.0017 (13)
C12	0.0579 (18)	0.116 (3)	0.0528 (18)	0.0025 (18)	0.0210 (14)	0.0104 (18)
N1	0.0386 (11)	0.0409 (13)	0.0508 (12)	0.0019 (10)	0.0214 (10)	0.0059 (10)
O1	0.0543 (11)	0.0385 (11)	0.0688 (12)	−0.0036 (8)	0.0259 (9)	0.0097 (8)
O2	0.0434 (9)	0.0596 (12)	0.0605 (11)	−0.0002 (8)	0.0300 (9)	−0.0021 (9)
O3	0.0552 (10)	0.0449 (11)	0.0555 (11)	−0.0038 (8)	0.0353 (9)	−0.0017 (8)
O4	0.0556 (11)	0.0447 (11)	0.0633 (11)	−0.0053 (8)	0.0391 (9)	0.0012 (8)
S1	0.0362 (3)	0.0394 (4)	0.0498 (4)	−0.0030 (2)	0.0202 (3)	0.0023 (3)
S2	0.0773 (5)	0.0750 (6)	0.0407 (4)	0.0063 (4)	0.0300 (4)	0.0059 (3)

### Geometric parameters (Å, °)

**Table:**

C1—S2	1.793 (4)	C6—S1	1.760 (3)
C1—H1A	0.960	C7—C9	1.378 (4)
C1—H1B	0.960	C7—H7	0.930
C1—H1C	0.960	C8—C10	1.375 (4)
C2—C3	1.508 (3)	C8—H8	0.930
C2—S2	1.801 (3)	C9—C11	1.385 (4)
C2—H2A	0.970	C9—H9	0.930
C2—H2B	0.970	C10—C11	1.381 (4)
C3—C4	1.538 (3)	C10—H10	0.930
C3—H3A	0.970	C11—C12	1.501 (4)
C3—H3B	0.970	C12—H12A	0.960
C4—N1	1.457 (3)	C12—H12B	0.960
C4—C5	1.509 (3)	C12—H12C	0.960
C4—H4	0.980	N1—S1	1.634 (2)
C5—O3	1.214 (3)	N1—H1	0.81 (3)
C5—O4	1.311 (3)	O1—S1	1.4238 (19)
C6—C7	1.378 (4)	O2—S1	1.4216 (18)
C6—C8	1.393 (4)	O4—H4A	0.820
			
S2—C1—H1A	109.5	C9—C7—H7	120.3
S2—C1—H1B	109.5	C6—C7—H7	120.3
H1A—C1—H1B	109.5	C10—C8—C6	119.3 (3)
S2—C1—H1C	109.5	C10—C8—H8	120.3
H1A—C1—H1C	109.5	C6—C8—H8	120.3
H1B—C1—H1C	109.5	C7—C9—C11	121.9 (3)
C3—C2—S2	113.29 (18)	C7—C9—H9	119.1
C3—C2—H2A	108.9	C11—C9—H9	119.1
S2—C2—H2A	108.9	C8—C10—C11	121.9 (3)
C3—C2—H2B	108.9	C8—C10—H10	119.1
S2—C2—H2B	108.9	C11—C10—H10	119.0
H2A—C2—H2B	107.7	C10—C11—C9	117.6 (3)
C2—C3—C4	113.7 (2)	C10—C11—C12	121.1 (3)
C2—C3—H3A	108.8	C9—C11—C12	121.3 (3)
C4—C3—H3A	108.8	C11—C12—H12A	109.5
C2—C3—H3B	108.8	C11—C12—H12B	109.5
C4—C3—H3B	108.8	H12A—C12—H12B	109.5
H3A—C3—H3B	107.7	C11—C12—H12C	109.5
N1—C4—C5	110.6 (2)	H12A—C12—H12C	109.5
N1—C4—C3	111.3 (2)	H12B—C12—H12C	109.5
C5—C4—C3	108.11 (18)	C4—N1—S1	119.67 (17)
N1—C4—H4	108.9	C4—N1—H1	119 (2)
C5—C4—H4	108.9	S1—N1—H1	108 (2)
C3—C4—H4	108.9	C5—O4—H4A	109.5
O3—C5—O4	125.0 (2)	O2—S1—O1	120.15 (11)
O3—C5—C4	122.6 (2)	O2—S1—N1	106.62 (12)
O4—C5—C4	112.3 (2)	O1—S1—N1	105.14 (11)
C7—C6—C8	119.9 (2)	O2—S1—C6	107.68 (11)
C7—C6—S1	120.36 (19)	O1—S1—C6	107.74 (12)
C8—C6—S1	119.7 (2)	N1—S1—C6	109.16 (11)
C9—C7—C6	119.5 (2)	C1—S2—C2	101.15 (15)
			
S2—C2—C3—C4	−179.45 (19)	C7—C9—C11—C10	1.1 (4)
C2—C3—C4—N1	70.7 (3)	C7—C9—C11—C12	−178.1 (3)
C2—C3—C4—C5	−167.6 (2)	C5—C4—N1—S1	123.41 (19)
N1—C4—C5—O3	17.0 (3)	C3—C4—N1—S1	−116.39 (19)
C3—C4—C5—O3	−105.1 (3)	C4—N1—S1—O2	48.6 (2)
N1—C4—C5—O4	−166.15 (18)	C4—N1—S1—O1	177.16 (17)
C3—C4—C5—O4	71.7 (3)	C4—N1—S1—C6	−67.5 (2)
C8—C6—C7—C9	−0.1 (4)	C7—C6—S1—O2	−16.2 (2)
S1—C6—C7—C9	176.96 (19)	C8—C6—S1—O2	160.89 (19)
C7—C6—C8—C10	0.2 (4)	C7—C6—S1—O1	−147.2 (2)
S1—C6—C8—C10	−177.0 (2)	C8—C6—S1—O1	29.9 (2)
C6—C7—C9—C11	−0.5 (4)	C7—C6—S1—N1	99.2 (2)
C6—C8—C10—C11	0.4 (4)	C8—C6—S1—N1	−83.7 (2)
C8—C10—C11—C9	−1.0 (4)	C3—C2—S2—C1	−70.4 (3)
C8—C10—C11—C12	178.1 (3)		

### Hydrogen-bond geometry (Å, °)

**Table:**

*D*—H···*A*	*D*—H	H···*A*	*D*···*A*	*D*—H···*A*
O4—H4A···O3^i^	0.82	1.85	2.672 (3)	174
N1—H1···O4^ii^	0.81 (3)	2.62 (3)	3.405 (3)	163 (2)
C4—H4···O1^iii^	0.98	2.25	3.165 (3)	155

Symmetry codes: (i) −*x*, −*y*+1, −*z*; (ii) *x*, *y*+1, *z*; (iii) *x*, *y*−1, *z*.
